# Sandwich-Like Fe&TiO_2_@C Nanocomposites Derived from MXene/Fe-MOFs Hybrids for Electromagnetic Absorption

**DOI:** 10.1007/s40820-020-0398-2

**Published:** 2020-02-18

**Authors:** Baiwen Deng, Zhen Xiang, Juan Xiong, Zhicheng Liu, Lunzhou Yu, Wei Lu

**Affiliations:** 1 Shanghai Key Lab. of D&A for Metal-Functional Materials, School of Materials Science & Engineering, Tongji University, Shanghai, 201804 People’s Republic of China; 2 School of Materials Science & Engineering, University of Shanghai for Science and Technology, Shanghai, 200092 People’s Republic of China

**Keywords:** MXene, Metal–organic frameworks, Nanocomposites, Electromagnetic wave absorption

## Abstract

Electromagnetic pollution has been causing a series of problems in people’s life, and electromagnetic absorbers with lightweight and broad absorbing bandwidth properties are widely desired. In this work, novel sandwich-like 2D laminated Fe&TiO_2_ nanoparticles@C nanocomposites were rationally designed and successfully developed from the MXene–MOFs hybrids. The formation of Fe and rutile-TiO_2_ nanoparticles sandwiched by the two-dimensional carbon nanosheets provided strong electromagnetic energy attenuation and good impedance matching for electromagnetic wave (EMW) absorption. As expected, the nanocomposites achieved a broad effective absorption bandwidth of 6.5 GHz at a thickness of only 1.6 mm and the minimum reflection loss (*RL*) value of − 51.8 dB at 6.6 GHz with a thickness of 3 mm. This work not only provides a good design and fabricating concept for the laminated metal and functional nanoparticles@C nanocomposites with good EMW absorption, but also offers an important guideline to fabricate various two-dimensional nanocomposites derived from the MXene precursors.

**Electronic supplementary material:**

The online version of this article (10.1007/s40820-020-0398-2) contains supplementary material, which is available to authorized users.

## Highlights

- Sandwich-like laminated Fe&TiO_2_@C nanocomposites were successfully prepared from the MXene–MOFs hybrids.
- A broad effective bandwidth of 6.5 GHz at a thickness of only 1.6 mm was achieved.

**Electronic supplementary material:**

The online version of this article (10.1007/s40820-020-0398-2) contains supplementary material, which is available to authorized users.

## Graphical abstract

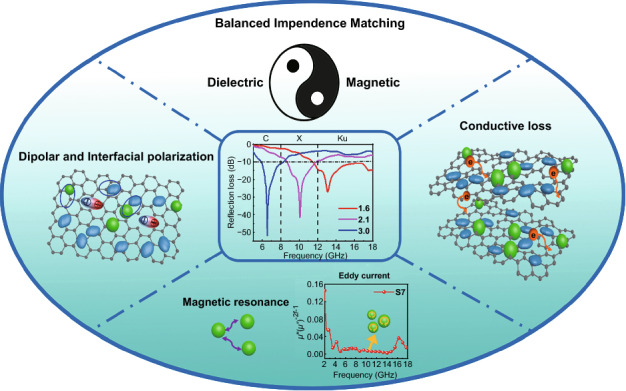

## Introduction

With the surging development of electrical communication devices, an extensive increase in hazardous electromagnetic radiation and interferes has become a severe problem in both civil and precision technical fields. Therefore, there is a great demand for developing broadband and high-efficiency electromagnetic wave (EMW) absorbing materials to minimize adverse effects in aspects of health and electrical devices [1–3]. Abundant efforts have been contributed to the development of high-efficiency EMW absorbers with a broad effective absorption bandwidth (EAB), especially for the radar frequency range of 2–18 GHz [4–6]. In general, materials with laminated structure are well known as promising EMW absorbers. Due to the unique layered structure of these laminated materials, which possess large surface area and heterogeneous interfaces, abundant dipolar, and interfacial polarizations could be generated during EMW absorption process. This is believed to be the main contribution to the excellent EMW absorbing ability [7–9]. Specifically, the most widely studied laminated EMW absorbers was the graphene-based two-dimensional materials in the past decade [2, 9–11]. Since the EMW absorption ability of graphene or reduced graphene oxide (RGO) nanosheets was not ideal, assembling functional particles with graphene or RGO has become a promising way to improve their performance, such as Ni/graphene [12], Co_3_O_4_–rGO [13], and ZnO–rGO [14].

MXene is a new family member of the big family tree of two-dimensional (2D) materials [15, 16], whose formula is *M*_*n*+1_*X*_*n*_*T*_*x*_, where *M* is an early transition metal, *X* is carbon and/or nitrogen, and *T*_*x*_ is the surface terminations usually −OH, –F, and/or –O [17–19]. Due to the laminated structure and unique combination of electrical conductivity and hydrophilicity, MXene is considered as an attractive candidate for various applications, including energy storage [16, 20, 21], sensors [22, 23], electromagnetic absorption, and electromagnetic interference shielding [3, 8, 24, 25]. Feng et al. [8] synthesized Ti_3_C_2_*T*_*x*_ nanosheets by HF system etching process, which appeared enhanced microwave absorption ability (*RL* value of − 40 dB at 2 mm thickness). Xu et al. [26] achieved a Ti_3_C_2_*T*_*x*_ with a *RL* value of − 41.9 dB at 13.4 GHz and thickness of only 1.1 mm by solvothermal treatment. Luo et al. [5] demonstrated a wide absorbing bandwidth (*EAB* over 10 GHz at a thickness of 3 mm) in Ti_3_C_2_*T*_*x*_ MXene absorbers. Ti_3_C_2_*T*_*x*_ is a representative of MXene family decorated with abundant active surface functional terminations [27–29], which could provide remarkable amount of dipolar and interfacial polarizations. Moreover, the variety surface terminations of MXene by ion intercalation [30], annealing [31], and oxidization [32] could offer tunable EMW absorbing properties. In general, EMW absorbers should have strong EM energy attenuation ability and moderate conductivity for impedance matching. However, the high electrical conductivity of Ti_3_C_2_*T*_*x*_ could be a big obstacle for the application as EMW absorbers [32]. Generally, the combination of magnetic particles and conductive carbon materials is considered as an effective way to achieve an improved EMW absorption by improving the impedance matching [33, 34]. Therefore, assembling the magnetic nanoparticles with the MXene oxides, such as TiO_2_/Ti_3_C_2_*T*_*x*_/Fe_3_O_4_ [6], NiO&TiO_2_@C [35], C/TiO_2_/*α*–Fe [36], and Co/TiO_2_@C [37], could effectively improve the impedance matching with little cost of its EMW energy attenuation performance. In summary, integrating magnetic particles with oxidized MXene could be a promising and effective way to obtain high-performance EMW absorption materials by optimizing the impedance matching.

The magnetic particles could be derived from novel metal organic framework (MOFs) materials [38–41]. Owing to their unique porous structures, MOFs are considered as ideal precursors for preparing nanostructured metal@C nanocomposites through facile heat treatment methods and provide intriguing electromagnetic (EM) absorption properties [42–45]. For instance, Zhou et al. [44] fabricated hierarchical MOF-derived Co/C@V_2_O_3_ hollow spheres with an enhanced EMW absorption performance (*RL*_min_ of − 40.1 dB and EBW of 4.64 GHz at a thickness of 1.5 mm). Liu et al. [46] synthesized porous carbon-wrapped Ni composites from Ni-based MOF with high EMW absorption performance (*RL*_min_ of − 51.8 dB and an EBW of 3.48 GHz at a thickness of 2.6 mm). Lü et al. [47] provided a porous Co/C nanocomposite derived from ZIF-67 (a kind of Co-based MOF), which achieved a broad EBW of 5.8 GHz with a *RL*_min_ value of − 35.3 dB. Li et al. [48] also successfully prepared hollow Co/C microspheres from ZIF-67, which exhibited a high *RL*_min_ of − 66.5 dB. Thus, the combination of 2D materials and MOFs derived magnetic particles could be a solution for achieving high-performance and broadband EMW absorbers. Though there are numerous works about introducing magnetic materials into MXene as mentioned, the EMW absorbers fabricated by in situ MXene–MOFs hybrids have been rarely studied [3, 37].

Herein, we demonstrate a facile route toward the rational construction of sandwich-like 2D laminated Fe&TiO_2_ nanoparticles@C nanocomposites derived from MXene/Fe-MOFs hybrids through a rapid microwave-assisted heating reaction followed by a suitable heat treatment. The in situ generated Fe and TiO_2_ nanoparticles embedded closely into each MXene-derived carbon nanolayers to form a two-dimensional sandwich-like laminated structure. The results indicate that the 2D laminated nanocomposites exhibit excellent tunable EMW absorption performance with high reflection loss (*RL*_min_ of − 51.8 dB), lightweight (a matching thickness of 1.6 mm), and broad bandwidth (6.6 GHz) due to the plentiful interfacial polarization and other mechanisms of EMW attenuation. Furthermore, our work offers an important facile approach to fabricate two-dimensional nanocomposites derived from MXene–MOF hybrids along with a good design and fabricating concept for the laminated metal and functional nanoparticles@C nanocomposites with good EMW absorption.

## Experimental Section

### Raw Materials

In this work, all chemical reagents were of analytical grade and not further purified. Specifically, hydrochloric acid (HCl), lithium fluoride (LiF), *N*,*N*-dimethylformamide (DMF), 1,4-benzenedicarboxylic acid (H_2_BDC), and ferric chloride hexahydrate (FeCl_3_·6H_2_O) were purchased from Shanghai Aladdin Industrial Corporation. Ti_3_AlC_2_ powders (> 98 wt% purity) were purchased from Laizhou Kai Kai Ceramic Materials Co., Ltd.

### Preparation of Ti_3_C_2_*T*_*x*_-MXene

Ti_3_C_2_*T*_*x*_ MXene was synthesized by removing Al atoms in Ti_3_AlC_2_ precursor based on the typical LiF–HCl etching method [19]. Firstly, 0.5 g of LiF was added into the concentrated 12 M HCl solution. Secondly, the solution was stirred 10 min with a magnetic Teflon stir to dissolve the LiF completely. Then, 0.5 g of Ti_3_AlC_2_ was slowly added into the solution and stirred at 50 °C for 24 h. The resultant suspension was washed by deionized water for several times via centrifugation process (3500 rpm, 5 min for every washing circle) until the pH value of supernatant became 5–6. Finally, the obtained Ti_3_C_2_*T*_*x*_ sediments were collected and dried in a vacuum oven for 24 h.

### Preparation of MXene–MOF Hybrids

The 0.12 g of as-prepared Ti_3_C_2_*T*_*x*_ MXene was added into 20 mL of N,N-dimethylformamide (DMF) and then sonicated for 30 min to form a uniform black solution. After that, 0.5405 g of FeCl_3_·6H_2_O was dissolved in the solution with sonication for 30 min. The 0.3323 g of terephthalic acid was dissolved into the Ti_3_C_2_*T*_*x*_ and Fe^3+^ mixture by vigorously stirring for 30 min. After that, 0.8 mL of 2 M NaOH solution was added dropwise into the mixture with ongoing stirring. Then, the as-prepared mixture was transferred into a 100-mL three-neck boiling flask. Following that, the reaction was conducted at 100 °C for 30 s in a microwave heating equipment. The obtained black solution was centrifuged and washed by DMF, deionized water, and ethanol. The resulting black product was dried in a vacuum oven for 3 h. The mass of resultant Ti_3_C_2_*T*_*x*_–Fe-MOFs hybrids was 0.26 g, indicating the mass ratio for Ti_3_C_2_*T*_*x*_ and Fe-MOFs was about 1.2.

### Preparation of Fe&TiO_2_@C Composites

The MXene–MOF hybrids obtained as above were carbonized at 700 °C for 2 h with a heating rate of 10 °C s^−1^ under a H_2_/Ar atmosphere. This product will be referred as S7. To ascertain the influences of structure modification on EMW absorption ability, the MXene–MOF hybrids were also carbonized at 600 and 800 °C under the same atmosphere condition, which will be referred as S6 and S8, respectively.

### Characterization

The thermogravimetric and differential scanning calorimetry (TG/DSC) curves of MXene–MOF hybrids were obtained to help understanding the transforming process from Ti_3_C_2_*T*_*x*_–Fe-MOF hybrids to Fe&TiO_2_@C composites, by using a thermal analysis system at the temperature range from 30 to 850 °C under N_2_ atmosphere. X-ray diffraction (XRD) patterns of the obtained S6, S7, S8, and Ti_3_C_2_*T*_*x*_ samples were recorded by X-ray diffractometer equipped with CuK_α_ radiation from 4° to 65° (a step scan of 0.02° and 1 s per step). Raman tests were carried out on a Raman microspectrometer with a He–Ne laser. The magnetic properties of the composites were measured by vibrating sample magnetometer (VSM, Lake Shore7307). The morphologies of the precursor and obtained powders were characterized by a transmission electron microscope (TEM) and a scan electron microscope (SEM).

To measure complex permittivity and complex permeability, paraffin was chosen as the matrix material due to its small contribution to dielectric and magnetic properties. S6, S7, and S8 samples were dispersed into the paraffin with loading ratio of 40 wt%. Finally, the well-mixed mixtures were molded into a torus-shape (3.04 mm of inner diameter and 7.00 mm of outer diameter) test samples. The complex permittivity and complex permeability were measured using an Agilent N5224A vector network analyzer in the frequency range from 2 to 18 GHz.

## Results and Discussion

### Synthesis of Fe&TiO_2_@C Nanocomposites

**Fig. 1:**
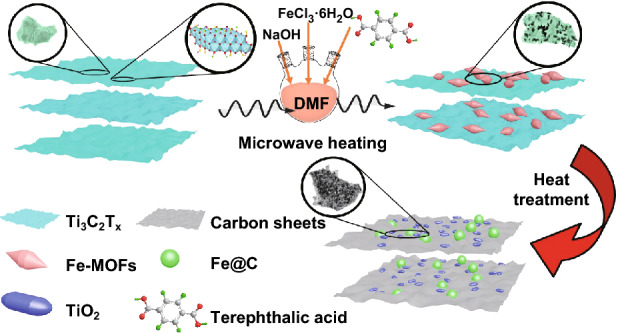
Schematic representation of the facile synthesis route of the Fe&TiO_2_@C by the EMW heating reaction followed by a heat treatment

The detailed fabrication process of the sandwich-like two-dimensional laminated Fe&TiO_2_ nanoparticles@C nanosheets composites is illustrated in Fig. 1. Firstly, the 2D laminated MXene nanosheets were synthesized based on a typical LiF-HCl etching method. Then, the Ti_3_C_2_*T*_*x*_–Fe-MOFs hybrids were facilely synthesized by the microwave-assisted heating process using FeCl_3_·6H_2_O, terephthalic acid, and NaOH as reactants in a DMF solution. During the reaction process, Fe^3+^ ions, which were inserted in or absorbed on the layers of Ti_3_C_2_*T*_*x*_, could form a shuttle-like Fe-MOFs by coordinating the carboxylate groups of terephthalic acid by strong metal–ligand bonds [49]. Finally, the sandwich-like two-dimensional Fe&TiO_2_@C nanocomposites were obtained via the carbonization of the Ti_3_C_2_*T*_*x*_–Fe-MOFs hybrids at high temperature for 2 h in H_2_/Ar atmosphere. In the carbonization process, shuttle-like Fe-MOFs have been transformed into Fe nanoparticles covered with carbon. At the same time, the TiO_2_ nanoparticles and laminated carbon nanosheets were in situ generated from the Ti_3_C_2_*T*_*x*_. Therefore, this transformation process provided a novel sandwich-like two-dimensional laminated structure of Fe&TiO_2_@C nanocomposites, in which the layered carbon nanosheets were filled with the Fe and TiO_2_ nanoparticles.

### Microstructure

**Fig. 2:**
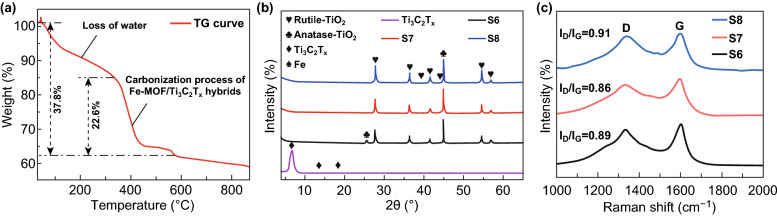
**a** TG curve of as-prepared Ti_3_C_2_*T*_*x*_–Fe-MOF hybrids. **b** XRD patterns of as-prepared Ti_3_C_2_*T*_*x*_, S6, S7, and S8. **c** Raman spectra results of S6, S7, and S8

The TG curve of the Ti_3_C_2_*T*_*x*_–Fe-MOF hybrids is shown in Fig. 2a, while the DSC result is exhibited in Fig. S1. Three significant weight loss processes were observed from the TG curve. The first process, ranging from 30 to 110 °C, could be considered as a weight loss process for the non-combined water in hybrids. The following weight loss at temperature range of 115 to 340 °C was attributed to the loss of the combined water and surface functional groups [32]. The total weight loss from 30 to 340 °C was 18.2%. Another weight loss occurred at the temperature range of 340 to 570 °C, which accompanied with an exothermic peak at 400 °C, might be attributed to the carbonization of Fe-MOF and Ti_3_C_2_*T*_*x*_. It was worth noting that a sharp exothermic peak was found at 566 °C in the DSC curve, which could be the result for phase transformation from anatase to rutile. The XRD patterns of the as-prepared Ti_3_C_2_*T*_*x*_ and Ti_3_C_2_*T*_*x*_–Fe-MOFs hybrids carbonized at 600, 700, and 800 °C are demonstrated in Fig. 2b. The typical peaks corresponding to the (002), (004), and (006) planes of Ti_3_C_2_*T*_*x*_ could be seen at 6.7°, 13.7°, and 18.7°, respectively, suggesting the successful synthesis of Ti_3_C_2_*T*_*x*_ MXene [19, 50, 51]. It was seen that a small peak at 25.74° appeared after carbonizing at 600 °C, which was corresponding to the (101) plane of anatase-TiO_2_ (JCPDS No. 75-1537). The other peaks at 27.7°, 36.3°, 41.5°, 54.5°, and 56.84° in the XRD pattern of S6 were assigned to the (110), (101), (111), (211), and (220) planes of the rutile-TiO_2_ (JCPDS No. 78-1510). Moreover, the peak at 44.9° was indexed to the (110) plane of Fe (JCPDS No. 87-0722). As shown in Fig. 2b, the anatase phase disappeared when the temperature increased over 700 °C, which agreed with the results reported in other works [31, 52, 53]. With the temperature increasing to 800 °C, there were only rutile-TiO_2_ and Fe phase remained in the sample. The phase fraction and averaged grain size of all the three samples (S6, S7, and S8) were calculated based on the XRD data by using Jade software with the WPF refinement, and the results are summarized in Table 1. The fractions of iron phase kept around 23% when the carbonization temperature increased from 600 to 800 °C, indicating the Fe element in Fe-MOFs was completely reduced and transformed into zerovalent metallic state by hydrogen. On the other hand, the sum of phase fractions of TiO_2_ in all the three samples was close to 75%, suggesting the Ti element in Ti_3_C_2_*T*_*x*_ sheets was in the form of TiO_2_. The averaged grain size of rutile-TiO_2_ increased from 40.1 to 57.6 nm with the increasing carbonizing temperature. The formation of rutile-TiO_2_ and the disappearance of anatase-TiO_2_ could be beneficial to the dielectric loss since the electromagnetic response of latter phase is worse than that of the former [53]. In addition, the XRD pattern of Ti_3_AlC_2_ is provided in Fig. S2. The Raman spectra of the three samples (S6, S7, and S8) are shown in Fig. 2c. The peaks, which corresponded to the characteristic D and G bands of carbon, appeared at 1343 and 1597 cm^−1^ in the Raman spectra of S6, S7, and S8. The intensities ratio between D and G (*I*_D_/*I*_G_) of S6, S7, and S8 was 0.89, 0.86, and 0.91, respectively. Generally, the descending value of *I*_D_/*I*_G_ indicates that the amorphous carbon transforms into the ordered graphite carbon [28]. On the one hand, the decreasing *I*_D_/*I*_G_ values for S6 to S7 samples could imply the improved carbon ordering with the increasing temperature. Normally, the amount of defects would be reduced with the increasing temperature, which led to the decreasing *I*_D_/*I*_G_ value and the transformation from amorphous carbon to ordered carbon [54]. On the other hand, the *I*_D_/*I*_G_ values showed an increasing tendency from S7 to S8. This result could be interpreted as being caused by an increase in the number, or in the size, of the graphitic domains [55–57]. It was worth mentioning that the increasing order of carbon is beneficial to increase the conductivity of carbon sheets, which could contribute to the conductive loss in EMW absorption process [35].

**Table 1:** Phase fraction and averaged grain size of S6, S7, and S8

Samples	Phase fraction (%)			Averaged grain size (nm)	
	Anatase	Rutile	Fe	Anatase	Rutile
S6	6.8 (0.8)	71.8 (4.8)	21.4 (1.4)	17.3	40.1 (9.6)
S7	**–**	75.2 (5.2)	24.8 (1.7)	**–**	52.4 (12.6)
S8	**–**	76.6 (5.3)	23.4 (1.6)	**–**	57.6 (11.5)

**Fig. 3:**
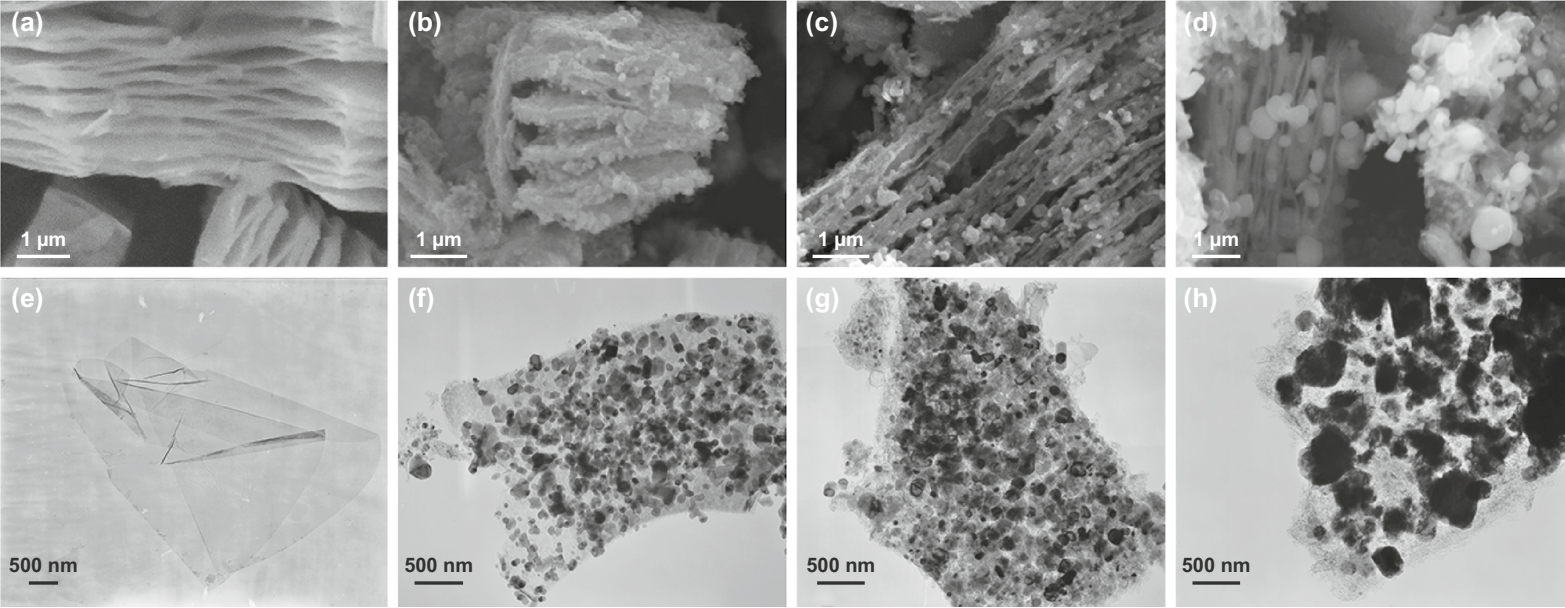
SEM and TEM images of Ti_3_C_2_*T*_*x*_ (**a**, **e**), S6 (**b**, **f**), S7 (**c**, **g**), and S8 **(e**, **h**)

The typical layered structure of Ti_3_C_2_*T*_*x*_ is clearly exhibited in Fig. 3a [51]. Additionally, the morphologies of S6, S7, and S8 observed by SEM are shown in Fig. 3b–d. The similar 2D laminated structure and small particles inserted between the layers were found in both S6 (Fig. 3b) and S7 (Fig. 3c). As for S8, it could be seen that the particles between layers grew significant and showed a high degree of aggregation in Fig. 3d. The EDS result for as-prepared Ti_3_C_2_*T*_*x*_ presented that the residual Al element was only 2.8 wt%, which confirmed the successful etching of Ti_3_AlC_2_, as shown in Fig. S4. The element mapping analysis for S7 further illustrated the uniform distribution of the Fe, Ti, C, and O elements in the sandwich-like 2D Fe&TiO_2_@C composites, as shown in Fig. S5. The TEM images of the Ti_3_C_2_*T*_*x*_, as-synthesized S6, S7, and S8 are presented in Fig. 3e–h. Before the carbonization process, it was seen that the shuttle-like Fe-MOFs, whose size was about 200–300 nm in length and 100–150 nm in width, were distributed homogenously in the laminated thin Ti_3_C_2_*T*_*X*_ sheets as shown in Fig. S3. After carbonating at 600 °C, the smooth Ti_3_C_2_*T*_*X*_ sheets were transformed into coarse disordered carbon sheets while rectangle TiO_2_ nanoparticles were precipitated. In addition, in our previous work, the microstructure evaluation of the Fe-MOFs during carbonization was studied [58]. Firstly, the Fe-MOFs transformed into Fe_3_O_4_ nanoparticles and porous carbon. Then, it began to collapse and transform into Fe nanoparticles with increasing the temperature. Due to the reduction atmosphere and relative longer carbonization time applied in this work, the Fe-MOFs decomposed into Fe nanoparticles wrapped by carbon, as shown in Fig. 3f. The Fe@C played an important role in improving impendence matching and providing interfacial polarization and magnetic loss. When the temperature increased to 700 °C, the size of the derived TiO_2_ and Fe nanoparticles slightly increased, and the aggregation of nanoparticles also became more intense (Fig. 3g). Furthermore, the TiO_2_ and Fe nanoparticles grew up excessively at 800 °C, which was consistent with the XRD results shown in Table 1, and ended up in oversized particles, as exhibited in Fig. 3h. This sandwich-like 2D laminated structure of Fe&TiO_2_@C composites was beneficial to the absorption of EM energy [37, 53].

### Electromagnetic Parameters and Microwave Absorption Performance

**Fig. 4:**
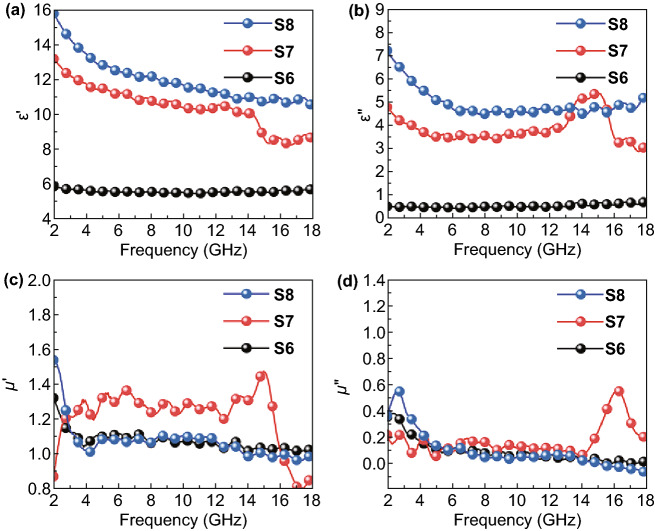
Frequency dependences of **a** real and **b** imaginary parts of complex permittivity, **c** real and **d** imaginary parts of complex permeability for the S6, S7, and S8 at the frequency range of 2–18 GHz

The electromagnetic absorption of Fe&TiO_2_@C composites was determined by the relative complex permittivity (*ε*_r_) and permeability (*μ*_r_). Normally, the real parts of permittivity and permeability (*ε*′ and *μ*′) represent for the storage ability of electromagnetic energy, while the imaginary parts of those (*ε*″ and *μ*″) stand for the energy dissipation. Figure 4a, b shows the complex permittivity of Fe&TiO_2_@C composites derived at different temperatures at the frequency ranging from 2 to 18 GHz. Both the real and imaginary parts of permittivity of Fe&TiO_2_@C composites were enhanced with the increasing carbonizing temperatures. In detail, the *ε*′ value of S6 maintained constant at around 6.0 within the whole frequency range, indicating the poor energy storage ability. However, S7 and S8 exhibited much higher value of *ε*′ than S6 (~ 12 for S7 and ~ 13 for S8), which probably were resulted from the disappearance of the anatase-TiO_2_ and the formation of more rutile-TiO_2_ when the carbonating temperature reached over 600 °C according to the XRD results. Besides, the mildly enlarged *ε*′ value of S7 and S8 within the frequency was also helpful for achieving excellent EMW absorption. Similarly, the *ε*″ value of S6 stayed around 0.5 while that of S7 declined from 4.7 to 3.5 and S8 slowly decreased from 7.2 and then mildly fluctuated at around 4.6. Compared with S6, the S7 and S8 with higher *ε*″ values exhibited stronger EMW attenuation ability. In addition, the *ε*′ and *ε*″ plots of S7 exhibited an obvious fluctuation with the frequency ranging from 12 to 16 GHz, revealing the existence of polarization behavior. As for S6, the low graphitization degree, which was identified by the Raman spectra results, caused the low values of permittivity leading to the flat *ε*′ and *ε*″ curves [59]. The *ε*′ and *ε*″ curves for S8 at the frequency range of 12–16 GHz were mildly fluctuated, which could be attributed to the aggregation of nanoparticles hindering the polarization process. It was reasonable to assume that these dielectric properties mainly come from the polarization process, which were induced by the defects, the heterointerfaces, and the conductive carbon sheets existed in the Fe&TiO_2_@C nanocomposites.

The magnetic hysteresis loops of all the three samples showed typical ferromagnetic features, as shown in Fig. S6a, b. The saturation magnetization (*M*_s_) of 19.7, 29.3, and 28.7 emu g^−1^ and coercivity (*H*_c_) of 72.7, 84.7, and 37.8 Oe were corresponded to the S6, S7, and S8, respectively. The *μ*′ and *μ*″ of the samples in the frequency range of 2–18 GHz are shown in Fig. 4c, d. The *μ*′ of S6 and S8 stayed at a value of 1.1 while S7 showed a relative higher value of 1.3 within the most of the measuring frequency range. In the frequency range of 2–6 GHz, some peaks for *μ*″ of S6 and S8 illustrated the existent of magnetic resonance behavior [60, 61]. Furthermore, the *μ*″ of S6 and S8 both decreased to nearly 0 when the frequency increased over 6 GHz, indicating the nature characteristic of the magnetic loss of S6 and S8 in the high frequency resulting from the Snoek′s limitation of Fe [62]. In addition, the S8 exhibited negative values of *μ*″ in the frequency range of 15–18 GHz, which was normally attributed to the radiation of magnetic energy [63–65]. According to Maxwell equations, this phenomenon could be caused by the magnetic energy released from internal magnetic field, which was induced by the motion of charges in carbon frameworks under the alternating EM field. Unlike the former ones, the *μ*″ plot of S7 changed barely in the frequency range of 2–14 GHz, and a peak appeared at 16 GHz which was also attributed to magnetic resonance in high frequency range [61]. According to the above analyses, the S7 sample might perform better impendence matching and electromagnetic energy dissipation ability than both S6 and S8.

**Fig. 5:**
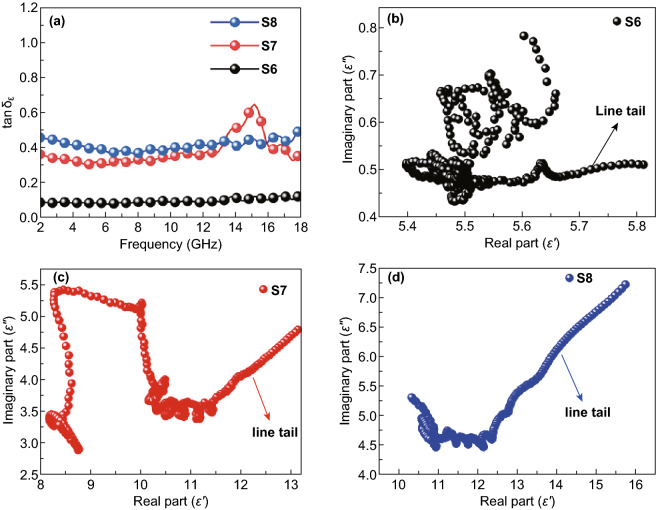
**a** Dielectric loss tangent of S6, S7, and S8 from 2 to 18 GHz. Plots of *ε*′ versus *ε*″ for **b** S6, **c** S7, and **d** S8

The dielectric loss tangent (tan*δ*_*ε*_ = *ε*″/*ε*′) of samples was calculated and is shown in Fig. 5a. Overall, the value of tan*δ*_*ε*_ showed an increasing trend with the increasing carbonization temperatures from 600 to 800 °C. This could also be attributed to the formation of rutile-TiO_2_ and defects when the treatment temperature increased over 600 °C. Generally, in the frequency range of 2–18 GHz, electronic polarization, Debye dipolar, and interfacial polarization and its related relaxation processes are the main sources of dielectric loss [9]. Firstly, the introduction of Fe nanoparticles, TiO_2_ nanoparticles, and carbon nanosheets could form multiple interfaces, such as Fe–C, TiO_2_–C and Fe–TiO_2_, to generate interfacial polarization and related relaxation process. The interfacial polarization process as mentioned could cause the Maxwell–Wagner effect [42, 66], which contributed to the enhanced dielectric loss of Fe&TiO_2_@C composites. The dipolar polarization was also one of the main factors to dissipate the EMW energy, since the Fe&TiO_2_ nanoparticles and defects distributed on the carbon sheets would form dipoles.

In order to further understand the polarization behavior during the EM absorption process, the plots of *ε*′ versus *ε*″ of all three samples are drawn in Fig. 5b–d. Based on the Debye relaxation, the relationship between *ε*′ and *ε*″ could be described as Eq. (1):

1 $$\left( {\varepsilon^{\prime} - \frac{{\varepsilon_{\text{s}} + \varepsilon_{\infty } }}{2}} \right)^{2} + \left( {\varepsilon^{\prime\prime}} \right)^{2} = \left( {\frac{{\varepsilon_{\text{s}} - \varepsilon_{\infty } }}{2}} \right)^{2}$$

where *ε*′ and *ε*″ represent the real and imaginary part of permittivity, while *ε*_s_ and *ε*_∞_ represent the static constant and the dielectric constant at infinite frequency, respectively. Hence, the semicircles in the plots of *ε*′ versus *ε*″ could be signs for the presence of polarization process. As shown in Fig. 5b, there were several semicircles in the plot of S6, indicating the existence of polarization process. As for S7, it could be seen from Fig. 5c that multiple semicircles present in the frequency range of 6–18 GHz, which suggested the polarization processes happened among most of the tested frequency range [36, 67]. The plot of S8 also exhibited a lot of small semicircles in the frequency range of 8–18 GHz as shown in Fig. 5d. In addition, the observed semicircles for S6, S7, and S8 mostly appeared in frequency range of 10–18 GHz, suggesting that the polarization and related relaxation process occupied the dominate position in dielectric loss mechanism at high frequency range. These polarization processes may be caused by the diploes and interfaces existed in the Fe&TiO_2_@C nanocomposites, which were beneficial to the EMW energy dissipation. Additionally, the conductive loss, which was mainly originated from the carbon nanosheets and Fe nanoparticles, could be confirmed by the presence of the line tails in the plots of *ε*′ versus *ε*″ [39, 42]. Furthermore, the conduction loss also plays an important role in EMW attenuation. As shown in Fig. 5b, c, the length of line tail extended as the temperature increased, indicating that the conductive loss was gradually strengthened. Therefore, the above results revealed the existence of three main factors including dipole polarization relaxation, interfacial polarization relaxation, and conductive loss, which were in favor of the dielectric loss.

**Fig. 6:**
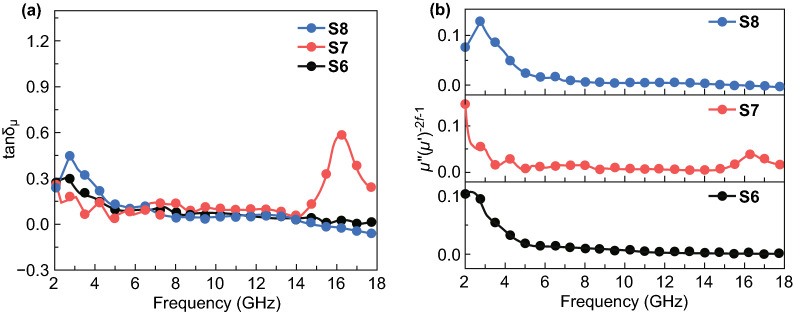
**a** Magnetic loss tangent of S6, S7, and S8. **b** The calculated *μ*′*(μ*″)^−2^*f*^−1^ for S6, S7, and S8 at the frequency range of 2–18 GHz

Besides the dielectric loss showing significant effects on EMW absorption, the magnetic loss also plays a key role in EMW absorption, which is theoretically originated from magnetic resonance, eddy current effect, and magnetic hysteresis [3, 9]. It is reasonable to exclude magnetic hysteresis loss for the applied weak and relative high-frequency electromagnetic field [6]. Normally, the magnetic resonance can be divided into natural resonance and exchange resonance. The natural resonance often happens in the low frequency range (lower than 10 GHz), while the exchange resonance takes place at over 10 GHz range [3]. The calculated magnetic loss tangent (tan*δ*_*μ*_ = *μ*″/*μ*′) of samples is shown in Fig. 6a. The tan*δ*_*μ*_ plots of S6 and S8 had a small peak at initial frequency range (2–4 GHz), which were assumed as a result of natural resonance, and they stayed around 0.05 in the frequency range of 5–18 GHz. As for the tan*δ*_*μ*_ plot of S7, it fluctuated mildly at low frequency range and kept around 0.05 in the range of 5–14 GHz. Then, the tan*δ*_*μ*_ plot of S7 exhibited a peak at high frequency range (14–18 GHz), whose apex exceeding 0.5 suggesting strong magnetic loss at related frequency range. Magnetic eddy current is another critical role for magnetic loss, which is inevitable in magnetic system. Such effect can be evaluated by *μ*″(*μ*′)^−2^*f*^−1^, where *μ*′ and *μ*″ represent the real and imaginary part of permittivity and *f* is the frequency of EM field, respectively. When there exists eddy current effect, the value of *μ*″(*μ*′)^−2^*f*^−1^ will be a constant as the frequency is changing [6, 36, 68]. Otherwise, there exists other magnetic loss mechanism in the system. As shown in Fig. 6b, the *μ*″(*μ*′)^−2^*f*^−1^ plots of S6 and S8 stayed as a constant from 8 to 18 GHz, and the *μ*″(*μ*′)^−2^*f*^−1^ value of S7 was a constant at the range of 6–14 GHz. These results confirmed that the magnetic loss of Fe&TiO_2_@C nanocomposites was mainly caused by eddy current effect within the tested frequency range. Additionally, the magnetic resonance, including natural and exchange resonance, also played an important role in magnetic loss based on the previous analysis of *μ*′ and *μ*″.

**Fig. 7:**
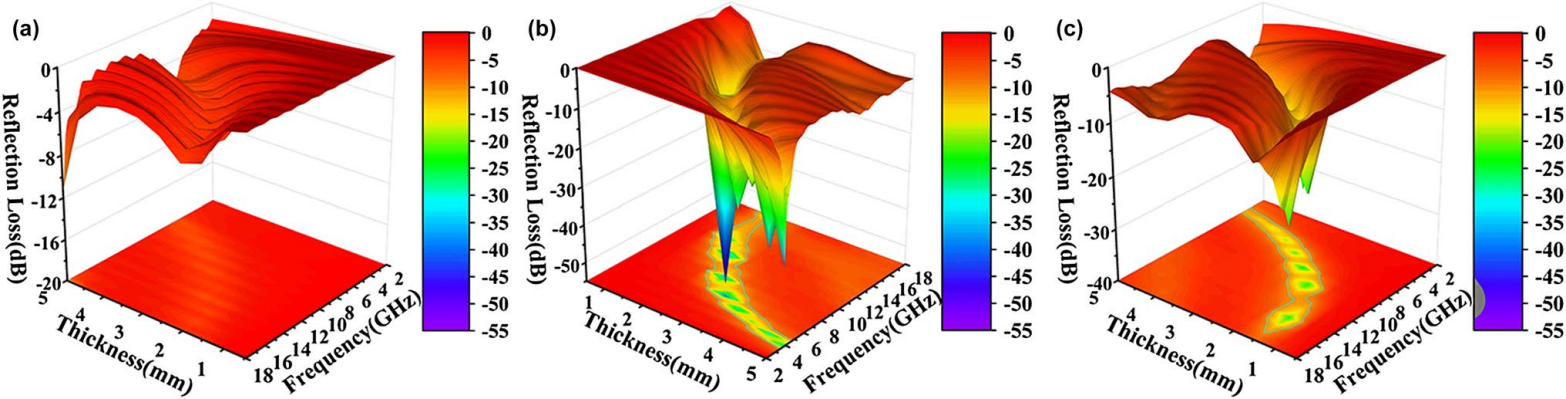
3D maps of *RL* values for S6 (**a**), S7 (**b**), and S8 (**c**) at different thicknesses over 2–18 GHz

The EM wave reflection loss (*RL*) values of the Fe&TiO_2_@C nanocomposites were calculated based on the measured data of EM parameters as Eqs. (2) and (3) [69–71]:

2 $$RL \ \left( {\text{dB}} \right) = 20\log \left| {\frac{{{\text{Z}}_{\text{in}} - {\text{Z}}_{0} }}{{{\text{Z}}_{\text{in}} + {\text{Z}}_{0} }}} \right|$$

where the input impedance *Z*_in_ of the absorber is given by the following equation:

3 $$Z_{\text{in}} = Z_{0} \sqrt {\mu_{\text{r}} /\varepsilon_{\text{r}} } \tanh \left| {j\left( {\frac{2\pi fd}{c}} \right)\sqrt {\mu_{\text{r}} \varepsilon_{\text{r}} } } \right|$$

where *ε*_r_, *μ*_r_, *f*, and d represent the complex permittivity and permeability of the EMW absorption materials, the related EM frequency, and the thickness of absorbers, respectively. Effective absorption bandwidth (*EAB*) is defined as the frequency range related to the *RL* value lower than − 10 dB, which indicates that more than 90% of the EM wave energy could be absorbed in this range. Figure 7a–c illustrates the 3D maps of *RL* data for S6, S7, and S8 at different thicknesses over 2–18 GHz. Besides, the *ε*′ and *ε*″ (Fig. S7a, b), *μ*′ and *μ*″ (Fig. S7c, d)), and *RL* results (Fig. S8) for Fe-MOFs and Ti_3_C_2_*T*_*x*_ (MXene) after carbonized at 700 °C are shown in Supporting Information as comparison. S6 showed a weak EMW absorption performance, which could be related to its low dielectric and magnetic loss, while a maximum *EAB* of 6.5 GHz (from 11.5 to 18 GHz) with a matching thickness of only 1.6 mm was achieved for S7 sample. It can be further tuned to own a strong EMW absorption ability by changing its thickness. For example, its *RL*_min_ could reach − 41.5 dB at 10.1 GHz with an *EAB* of 3.2 GHz when thickness is 2.1 mm, and a − 51.8 dB *RL*_min_ (at 6.6 GHz) could be obtained at thickness of 3.0 mm. Despite that the dielectric loss of S7 was not the biggest comparing with that of S6 and S8, it achieved a better EMW absorption performance than S8 sample, which was ascribed to the more uniform distribution of Fe and TiO_2_ nanoparticles on the laminated carbon surfaces, leading to a better synergistic effect of dielectric and magnetic response [35–37]. In the case of S8, it also showed a relatively wide *EAB* of 5.0 GHz (from 12 to 17 GHz) with thickness of 1.6 mm, as shown in Fig. 7c. Though the S8 performed bigger dielectric loss and similar magnetic loss compared with S7, its EMW absorption performance was not ideal, since EMW absorption needs the synergy effort of impendence matching and attenuation ability for EM energy. In order to compare the EMW absorption performance obtained in current investigation and studies on MXene-based materials, the related results are summarized in Table 2. It is shown that the Fe&TiO_2_@C composite derived at 700 °C owned tunable, lightweight, and broadband EM absorption performance compared with the other MXene and related materials reported in the studies.

**Table 2:** EMW absorption performance of MXene-based materials

Material	Loading ratio (wt%)	*RL* < − 10 dB		*RL*_min_		References
		Bandwidth (GHz)	d (mm)	Value (dB)	d (mm)	
Ti_3_C_2_*T*_*x*_	50	6.8	2	− 40	3	[8]
Ti_3_C_2_*T*_*x*_-24	55	5	1.7	− 42.5	1.7	[72]
Ti_3_C_2_*T*_*x*_	55	2.8	1.8	− 30	1.8	[32]
Annealed Ti_3_C_2_*T*_*x*_	50	2.8	1.85	− 48.4	1.7	[32]
TiO_2_/C	45	5.6	1.7	− 36	1.7	[53]
TiO_2_/Ti_3_C_2_*T*_*x*_/Fe_3_O_4_	–	2	1.9	− 57.3	1.9	[6]
C/TiO_2_/*α*–Fe	70	3.5	1.5	− 45.1	3.5	[36]
Co/TiO_2_–C	45	4.6	2	− 41.1	3	[37]
Fe&TiO_2_@C	40	6.5	1.6	− 51.8	3	Our work

**Fig. 8:**
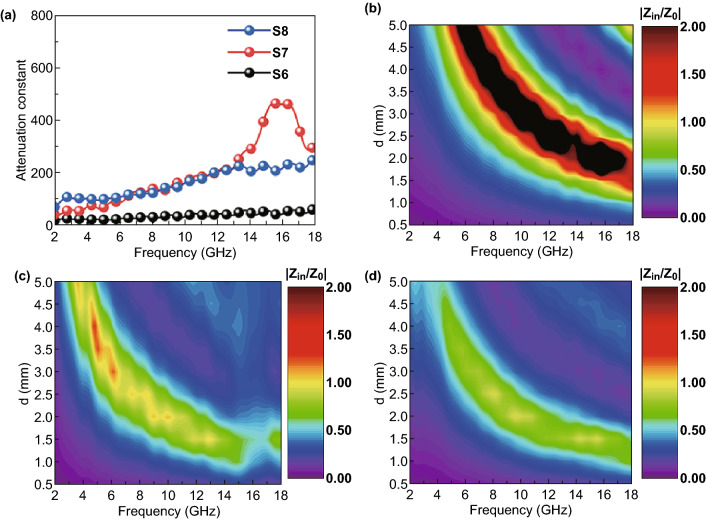
**a** Attenuation constants results of S6, S7, and S8. 2D contour maps of |*Z*_in_/*Z*_0_| at different thicknesses from 2 to 18 GHz for **b** S6, **c** S7, and **d** S8

Generally, two indispensable factors should be taken into consideration while aiming to achieve outstanding EMW absorption properties. The first is EMW attenuation capability of the absorption materials, and the second is the impedance matching. Normally, attenuation constant is used to qualify the EM energy attenuation ability of absorbers. Attenuation constants *α* of the samples, which are shown in Fig. 8a, can be calculated by Eq. (4) [67]:

4 $$\alpha = \frac{\sqrt 2 \pi f}{c}\sqrt {\left( {\mu^{\prime\prime}\varepsilon^{\prime\prime} - \mu^{\prime}\varepsilon^{\prime}} \right) + \sqrt {\left( {\mu^{\prime\prime}\varepsilon^{\prime\prime} - \mu^{\prime}\varepsilon^{\prime}} \right)^{2} + \left( {\mu^{\prime\prime}\varepsilon^{\prime} + \mu^{\prime}\varepsilon^{\prime\prime}} \right)^{2} } }$$

Both S7 and S8 showed strong EMW attenuation capability in the high frequency range (12–18 GHz), while S6 exhibited a weak performance in the whole tested range (2–18 GHz). It is obviously that S7 possessed the best attenuation capability compared to the other samples, which was beneficial to enhance EM wave energy attenuation. The uniform distribution of the Fe and TiO_2_ nanoparticles and relative high graphitization degree of carbon sheets are the reasons for the high EM energy dissipation capability of S7. Conversely, the poor graphitization and formation of anatase-TiO_2_ nanoparticles in S6 prevented it to achieve a high attenuation constant. The normalized characteristic impedance (*Z* = |*Z*_in_/*Z*_0_|) is used to evaluate the impedance matching of the samples, which is calculated based on Eq. (3). Normally, a good impedance matching means that the EMW could successfully enter the absorbers. To take sample thickness and measuring frequency into consideration, the 2D contour maps of *Z* for S6, S7, and S8 are exhibited in Fig. 8b–d. As is well known, achieving good impedance matching requires that the value of *Z* is equal or close to 1 [73, 74]. In Fig. 8b–d, the color of yellow represents the area in which *Z* equals 1, which representing the excellent impedance matching. The mismatch of impedance in S6 was apparent since the appearance of large black area, as shown in Fig. 8b. Compared with S6, the impedance matching for S7 and S8 showed a greatly improvement. Additionally, the yellow area of S7 was larger than that of S8, implying that S7 possessed a better impedance matching than S8. Overall, the S7 owned stronger EM energy attenuation ability and better impedance matching than the other two samples, leading to lightweight and broadband EM absorption performance of S7.

**Fig. 9:**
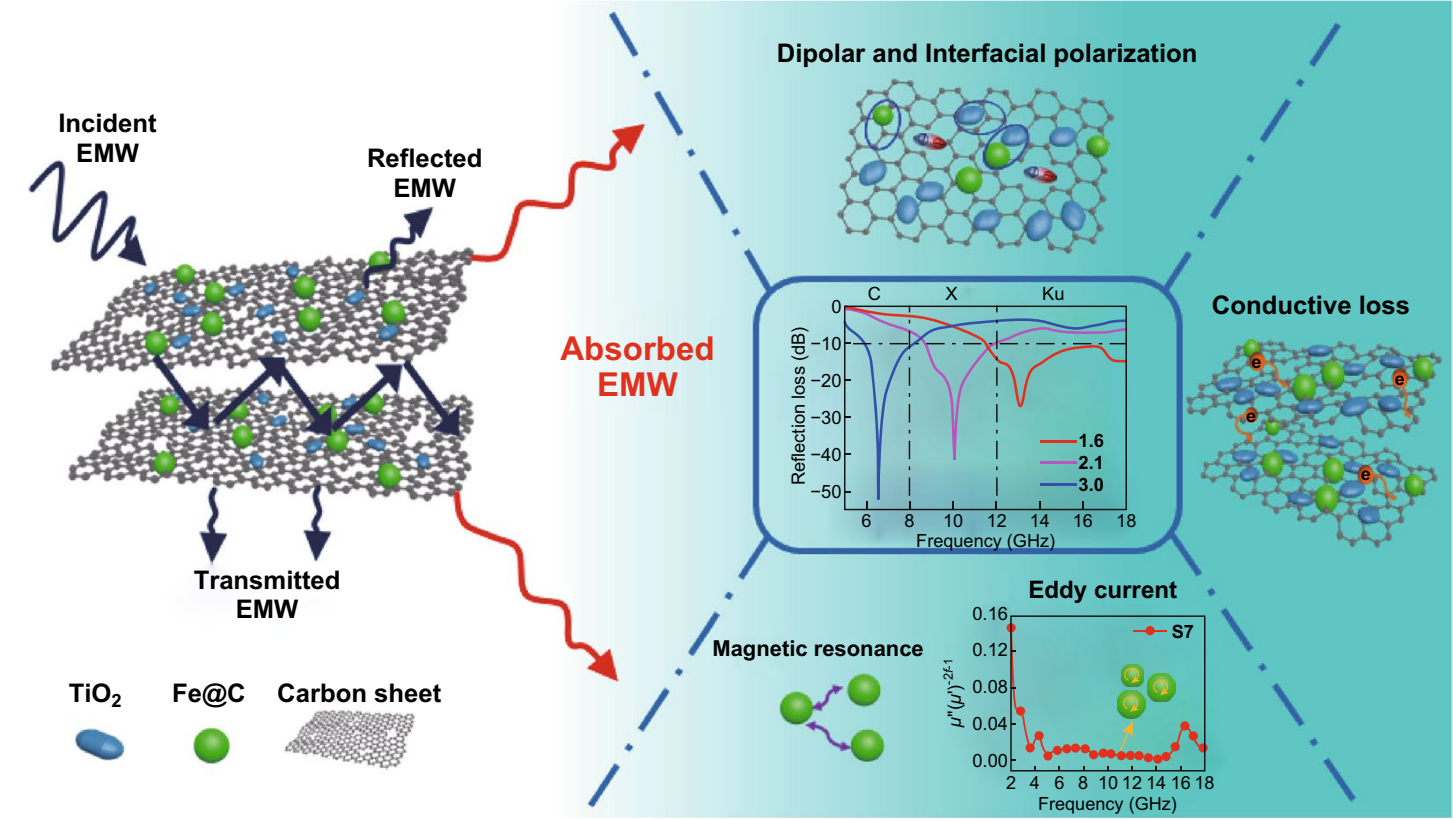
Illustration of EMW absorption mechanisms for Fe&TiO_2_@C nanocomposites

A possible EMW absorption mechanism for the sandwich-like laminated Fe&TiO_2_@C nanocomposites is illustrated in Fig. 9. Firstly, the multiple reflections between the conductive carbon layers and scattering among the Fe and TiO_2_ nanoparticles had taken place as soon as the EMW penetrated in the absorbers. These phenomena would be helpful in enhancing the EMW absorption efficiency. Additionally, the electron migration process including crossing and hoping over the conductive carbon sheets also played an important role in the EM energy attenuation. Secondly, dielectric loss produced by the polarization of dipoles and interfaces among the Fe, TiO_2_, and carbon would give a great contribution to transform the EM energy into thermal energy. In detail, the Fe, TiO_2_ nanoparticles, and carbon sheets with different conductivity could form capacitor-like structure, such as the contact positions of carbon sheets, the interfaces of Fe–TiO_2_, Fe–C, and TiO_2_–C. These capacitor-like structures could give rise to the accumulation and rearrangement of space charge, resulting in generating vast interface polarization processes in alternating EM field [3, 75, 76]. Thus, the interfacial polarization played a main role in the EM attenuation process for this sandwich-like Fe&TiO_2_@C ternary nanocomposite. Moreover, the magnetic loss caused by natural resonance, exchange resonance, and eddy current were inclined to enhance the EM energy dissipation ability of the laminated Fe&TiO_2_@C nanocomposites. In addition, the improved impedance matching was achieved by introducing the rutile-TiO_2_ and magnetic Fe nanoparticles through the in situ formation of Fe-MOFs followed by a suitable heat treatment. Based on the above discussion, it is highly expected that the laminated Fe&TiO_2_@C nanocomposites could be a good candidate for high-performance EMW absorbing materials.

## Conclusion

In summary, sandwich-like 2D laminated Fe&TiO_2_@C nanocomposites with tunable EMW absorption performance have been synthesized from the MXene/MOFs precursors by the rapid microwave heating process followed by the carbonization process. The formation of Fe and rutile-TiO_2_ nanoparticles sandwiched by the two-dimensional carbon nanosheets provided strong electromagnetic energy attenuation and good impedance matching for EMW absorption. The Fe&TiO_2_@C nanocomposites exhibited a broad effective absorption bandwidth of 6.5 GHz (from 11.5 to 18 GHz) at a thickness of only 1.6 mm and the minimum *RL* value of − 51.8 dB at 6.6 GHz at a thickness of 3 mm, respectively. This work not only provides a good design and fabricating concept for the laminated metal and functional nanoparticles@C nanocomposites with good EMW absorption, but also offers an important guideline to fabricate various two-dimensional nanocomposites derived from the MXene precursors.

## Electronic supplementary material

Below is the link to the electronic supplementary material.

Supplementary material 1 (PDF 631 kb)
