# Supplementary material for: Sandwich-Like Fe&TiO2@C Nanocomposites Derived from MXene/Fe-MOFs Hybrids for Electromagnetic Absorption
Source: Nanomicro Lett. 2020 Feb 18;12:55. doi: 10.1007/s40820-020-0398-2 (PMC7770802; doi:10.1007/s40820-020-0398-2)
Supplement: Supplementary file 1 — Supplementary material 1 (PDF 631 kb) [file 40820_2020_398_MOESM1_ESM.pdf]

Supporting Information for

Sandwich-like Fe&TiO_2@C Nanocomposites Derived from MXene/Fe-MOFs Hybrids for Electromagnetic Absorption

Baiwen Deng[1], Zhen Xiang[1], Juan Xiong[1], Zhicheng Liu[1], Lunzhou Yu[2], Wei Lu[1, *]

[1]Shanghai Key Lab. of D&A for Metal-Functional Materials, School of Materials Science & Engineering, Tongji University, Shanghai 201804, People's Republic of China

[2]School of Materials Science & Engineering, University of Shanghai for Science and Technology, Shanghai 200092, People's Republic of China

*Corresponding author. E-mail: weilu@tongji.edu.cn (Wei Lu)

Supplementary Figures

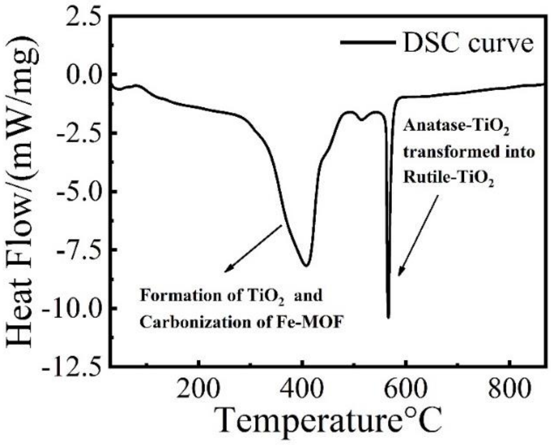

Fig. S1 DSC curve of $Ti_3C_2T_x$-FeMOF hybrids

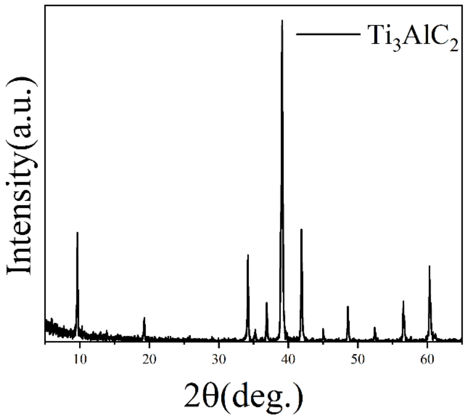

Fig. S2 XRD pattern of Ti_3AlC_2

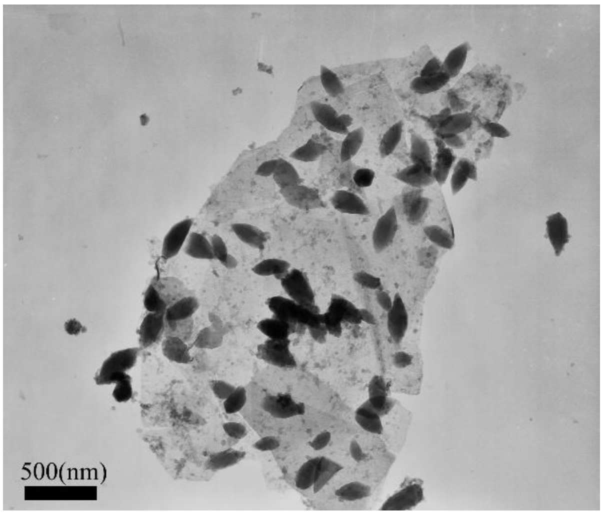

Fig. S3 Morphologies of $Ti_3C_2T_x$ MXene and $Ti_3C_2T_x$-FeMOF hybrids

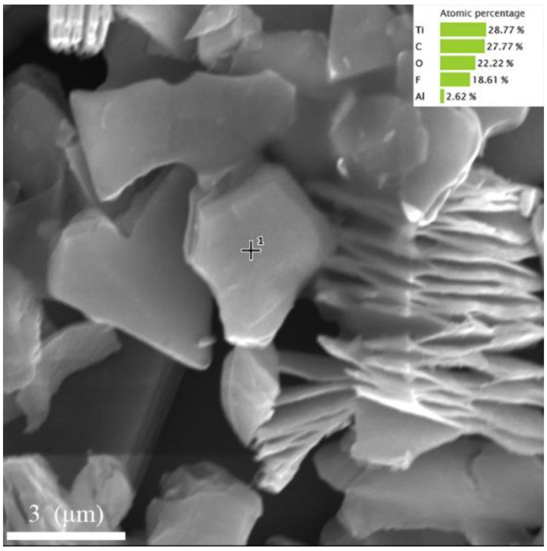

Fig. S4 EDS results of as-prepared $Ti_3C_2T_x$

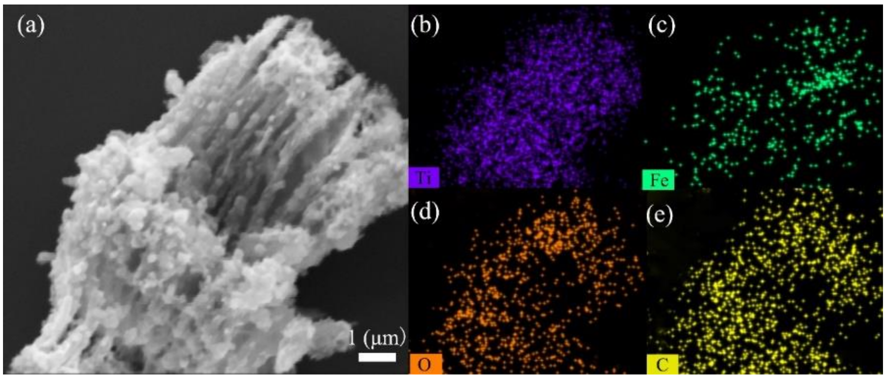

Fig. S5 Element mapping result of S7

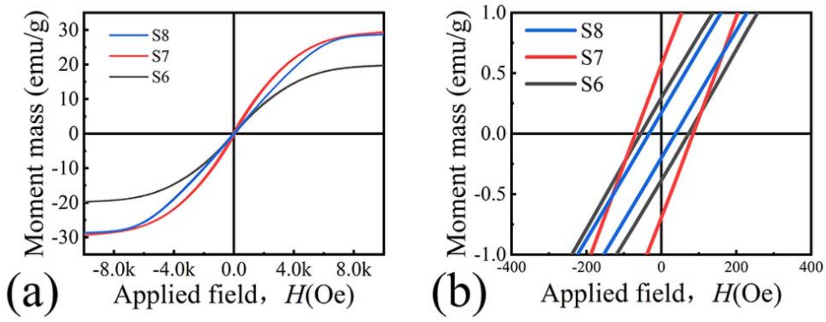

Fig. S6 a, b Magnetic hysteresis loops for S6, S7, and S8

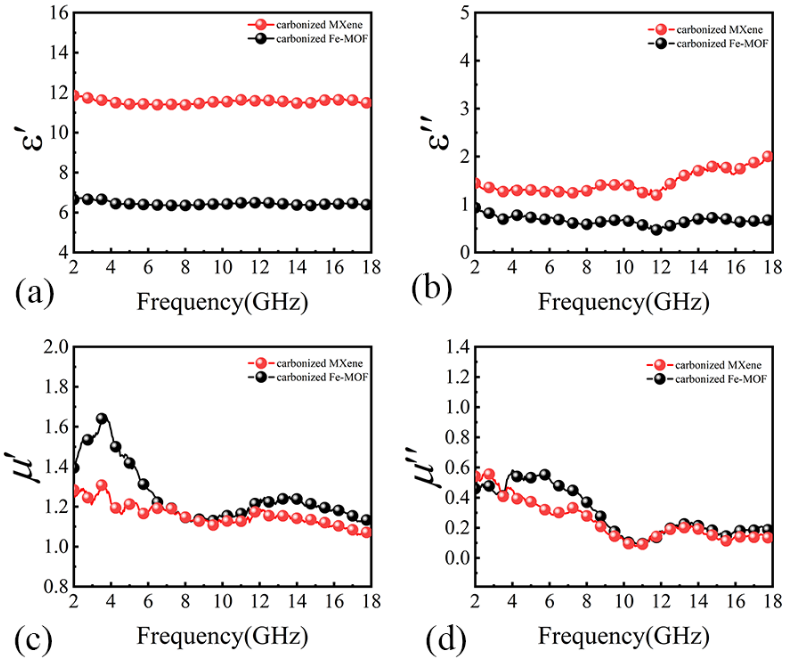

Fig. S7 Frequency dependences of **(a)** real and **(b)** imaginary parts of complex permittivity, **(c)** real and **(d)** imaginary parts of complex permeability for Fe-MOF and $Ti_3C_2T_x$ after carbonized at 700 °C at the frequency range of 2-18 GHz

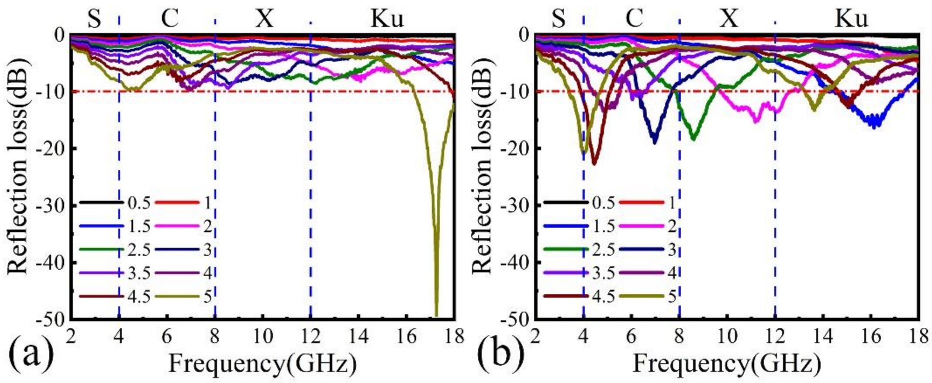

Fig. S8 *RL* results at different thickness for Fe-MOF **(a)** and $Ti_3C_2T_x$ **(b)** after carbonized at 700 °C
